# Knowledge, Awareness, and Practice of Safety and Emergency Response Among Scuba Divers in Malaysia: Questionnaire Development and Validation

**DOI:** 10.7759/cureus.53521

**Published:** 2024-02-03

**Authors:** Aladin Muhammad, Wan Mohd Zahiruddin Wan Mohammad, Siti Rabiatull Aisha Idris, Ahmad Filza Ismail

**Affiliations:** 1 Community Medicine, School of Medical Sciences, Universiti Sains Malaysia, Kubang Kerian, MYS; 2 Mechanical and Automotive Engineering Technology, Universiti Malaysia Pahang Al-Sultan Abdullah, Pekan, MYS

**Keywords:** content validity, face validity, reliability and validity, scuba divers, questionnaire development and validation

## Abstract

Introduction: Safety has become paramount to ensure that scuba diving continues to expand into new markets, with minimal risks, and that scuba diving translates into a safe and relaxed adventure in response to growing market demands. Research focusing on assessing the knowledge, awareness, and practices (KAP) regarding safety and emergency response among scuba divers has been limited, and there is a need for validated assessment tools in this area.

Methods: This study involved 555 scuba divers in Malaysia, and the questionnaire underwent a thorough development and validation process, including content and face validity assessments, as well as exploratory factor analysis. The validation of the knowledge domain was conducted using Item Response Theory (IRT) analysis, while awareness and practice were validated through exploratory and confirmatory factor analyses (EFA and CFA).

Results: The content validity of the instrument was confirmed, with all items scoring over 80% for Item Content Validity Index (I-CVI), Scale Content Validity Index (S-CVI), and Scale Content Validity Index/Average (S-CVI/AVE). The initial draft comprises three domains: knowledge, awareness, and practice. Knowledge items were analyzed using IRT and demonstrated acceptable difficulty and discrimination levels. For the awareness and practice domains, EFA showed a Kaiser-Meyer-Olkin measure (KMO) of 0.83 and 0.79, respectively, with a significant Bartlett's test of Sphericity (P < 0.001). EFA yielded three factors for both awareness and practice domains with a total of 12 items for awareness and 13 items for practice, with satisfactory factor loadings (≥0.3). The final model of CFA showed good fits for both awareness and practice domains in terms of absolute, parsimonious, and comparative measures. The composite reliability of awareness was acceptable with Raykov's rho of 0.71, whereas the practice domain fell slightly below the acceptable threshold at 0.55. This was attributed to low factor loading and a limited number of items within each factor. The final questionnaire now consists of 20 items for knowledge, 10 items for awareness, and 12 items for the practice domain.

Conclusion: The results of this validation and reliability study indicate that the newly developed questionnaire possesses favorable psychometric properties for assessing KAP related to safety and emergency response in the scuba diving context. This research is essential for harmonizing the perspectives of crucial stakeholders within the recreational scuba diving industry.

## Introduction

Recreational scuba diving unlocks the captivating mysteries of the ocean, allowing adventurers to immerse themselves in an enchanting underwater realm with over 128,000 certified divers and 6,600 diving centers worldwide.

The soaring popularity of scuba diving destinations is transforming marine resources into flourishing exploration havens. Safety remains paramount for expanding scuba diving into new markets [1]. Although the activity is relatively safe, awareness of potential hazards is crucial as residual risks persist and recreational scuba diving operations encounter challenges that add to the excitement [2]

Recreational scuba diving in the US and Canada has a high casualty fatality rate, with 2,046 diving-related injuries reported in 2014. The most common injuries are barotrauma, decompression illness, and marine envenoming [3]. Malaysia reports about 20 diving incidents annually, with accidents proportional to the number of divers [4]. However, there needs to be more updated literature on diving-related injuries in Malaysia over the past few decades.

The safety of scuba diving hinges on self-responsibility; therefore, knowledge, awareness, and practice (KAP) on risk and accident-preventing behaviors are essential; however, some divers engage in hazardous practices and disregard safety procedures [2]. Promoting a safety-first mentality is crucial for ensuring a safe diving experience. Despite numerous studies, a comprehensive KAP on safety and emergency response questionnaire still needs to be improved worldwide.

The lack of a thorough KAP questionnaire on safety and emergency response among recreational scuba divers worldwide underlines the need for a new questionnaire to be developed. This questionnaire can be used as a reliable instrument to assess the KAP level regarding safety and emergency response among scuba divers, providing a holistic perspective on this fundamental subject. With this pertinent data, stakeholders can take appropriate measures to reduce the number of casualties and improve safety practices in the scuba diving industry by bridging this gap.

## Materials and methods

The overall questionnaire development and validation occurred in two stages: 1) Questionnaire development and 2) Questionnaire validation process. The validation stage involved Item Response Theory (IRT) for the knowledge domain, while Exploratory Factor Analysis (EFA) and Confirmatory Factor Analysis (CFA) were employed for the awareness and practice domains.

Stage I: questionnaire development

The initial process includes a comprehensive literature search to identify available resources on KAP and relevant items and scales in existing safety and emergency response questionnaires.

The questionnaire comprises the proforma checklist (sociodemographic characteristics and diving profile) and the KAP questions (knowledge, awareness, and practice domain). Each KAP domain was subsequently divided into two main subdomains: safety and emergency response.

The knowledge domain focuses on safety procedures, equipment, and volume changes, while emergency response questions assess diving-related illnesses, early treatment methods, and emergency response plans. Meanwhile, most of the literature selected the factors domain from the theoretical framework by Morgan et al. [5] and Koo et al. [6] for the awareness and practice domain. The domain framework combined other theories, such as the health belief model, outcome-expectancy theory, and the Theory of Planned Behavior. This framework was used in the development of safety behavior change questionnaires.

The first version of the questionnaire went through a content validation process by six experts: three Dive Masters, and one each of Emergency Physician, Diving Medical Officer, and Occupational Health Doctor. A content validation form was established to specify the concept and allow experts to submit opinions to produce the second version of the questionnaire. Item-level content validity index (I-CVI), scale-level content validity index (S-CVI), and scale-level content validity index averaging calculation method (S-CVI/Ave) were calculated manually. A new tool should achieve at least 80% or higher agreement for acceptable content validity [7]. A few relevant amendments regarding the questions were applied during this process. Panel members refine items, compile raw ratings, and allow free-text comments. Quantitative data and expert input enhance questionnaire content validity and functionality.

The second version was then subjected to face validation by 10 intended respondents. Ten scuba divers were interviewed to assess the ability of a layperson to comprehend the questionnaire and to determine the relevance of the questions to the target audience. The raw panel rating will be compiled and calculated for comprehensibility and clarity of each item-level face validity index (I-FVI). Again, the questions that achieved at least 80% or higher agreement were acceptable face validity [7]. Based on the results of the face validation, an improved third version of the questionnaire has been designed for use in the remaining phases of the study.

The main parts, domains and subdomains, measurements, as well as the choices of response in the questionnaire are shown in Table 1.

**Table 1:** The first version of the knowledge, awareness, and practices (KAP) questionnaire on safety and emergency response among scuba divers

Part	Domain	Subdomain	Measurements	No of item	Scale
I	Proforma checklist	Sociodemographic characteristic, diving profile	Sociodemographic characteristic, diving profile	25	Open-ended, close-ended, multiple choice
II	Knowledge	Safety	Safety procedures, equipment, volume changes, physical changes	11	Yes/No/Not sure. 1 = correct answer, 0 = wrong/Not sure
II	Knowledge	Emergency response	Diving-related illnesses, early treatment methods, and emergency response plans	10	Yes/No/Not sure. 1 = correct answer, 0 = wrong/Not sure
II	Awareness	Safety	Benefits of compliance with safety instructions, environmental and equipment failure risk	10	1 = Strongly disagree, 2 = Disagree, 3 = Not sure, 4 = Agree, 5 = Strongly agree
II	Awareness	Emergency response	Importance of emergency action/assistance plans (dive center and personal)	6	1 = Strongly disagree, 2 = Disagree, 3 = Not sure, 4 = Agree, 5 = Strongly agree
II	Practice	Safety	Predive checklist, buddy system, refresher dive	10	1 = Never, 2 = Occasionally, 3 = Sometimes, 4 = Frequently, 5 = Every time
II	Practice	Emergency response	Ability to cope with accidents, personal diving response plan	6	1 = Never, 2 = Occasionally, 3 = Sometimes, 4 = Frequently, 5 = Every time

Stage II: questionnaire validation

The questionnaire validation process included IRT for the knowledge domain and EFA and CFA for the awareness and practice domains. From December 2022 to March 2023, the research was conducted at a few selected diving centers in Semporna and Kota Kinabalu, Sabah. For IRT and EFA, we enlisted 222 participants. Meanwhile, CFA involved 333 participants. The inclusion criteria for the study include individuals aged 18 years and older, those able to understand English, holders of a dive certificate at any level, and recreational divers. Meanwhile, divers with no history of diving after obtaining certification were excluded from the study. The participants were recruited using a universal sampling method. A brief explanation of the study was given to the participants, and informed consent was obtained from those who agreed to participate. The self-administered KAP questionnaire forms were distributed to each participant as a physical copy or a Google form. Statistical Package for Social Sciences (SPSS) version 26 (IBM Corp., Armonk, NY, USA) and R software version 4.0.5 (R Foundation for Statistical Computing, Vienna, Austria) were used to analyze the data.

i) Item Response Theory

IRT was used to analyze the knowledge domain. The optimal sample size for two-parameter logistic (2-PL) IRT has been estimated to be between 100 and 500 [8,9]. As a result, for this study, 222 participants were collected at 10% of the expected drop-off rate. The output for the knowledge domain was set to dichotomous as either correct or incorrect. The 2-PL IRT analysis using the “ltm” package version 1.2.0 was used to analyze the knowledge domain.

The difficulty levels of the knowledge domain items were classified as "Easy" (difficulty ≤ -2.0), "Average" (difficulty between -2.0 and 2.0), and "Hard" (difficulty > 2.0). However, in practice, the values typically fall within the range of -3 to +3 [10,11]. A discrimination value of less than 0.25 was regarded as unsatisfactory. In contrast, values equal to or more than 0.25 were deemed appropriate for measuring the item's capacity to distinguish between individuals with varying levels of expertise [12]. This classification aided in selecting a broad set of items that effectively tested participants' knowledge across different degrees of difficulty and ensured good discrimination between persons with varying levels of knowledge. The chi-square goodness-of-fit per item determined the fit of each item. Simultaneously, multidimensional item response theory was explored using the "mirt" package version 1.38.1 to assess the model’s goodness. Browne and Cudeck [13] proposed an acceptable Root Mean Square Error of Approximation (RMSEA) value of 0.08 or less.

ii) Exploratory Factor Analysis

To ascertain a sufficient sample size for EFA, we followed the recommendations of Comrey and Lee [9], who suggest 200 samples as fair sample size for EFA. So, for this study, 222 participants were recruited including a 10% dropout rate. The Kaiser-Meyer-Olkin (KMO) and Bartlett's sphericity test were used to determine sampling adequacy. The KMO value was greater than 0.5 [14], and Bartlett's significant test (p<0.001) was needed for the sample to be considered sufficient [15]. It is recommended that one or two factors should be retained to be adequate for an optimal balance between comprehensiveness and parsimony based on the combination of parallel analysis and minimum average partial, with scree plot as a potentially useful adjunct [16-18]. Meanwhile, the primary axis factoring method was used for the factor extraction. Promax oblique rotation with Kaiser normalization was selected because it honors the ubiquity of intercorrelations among social science variables [19]. Items were determined to have an acceptable loading factor greater than or equal to 0.30 [9,20-22]. Model fit will be indicated by a root mean square residual (RMSR) value of 0.05 or smaller and no individual residual coefficient greater than 0.10 [23].

iii) Confirmatory Factor Analysis

According to Comrey and Lee [9], a sample size of 300 is appropriate for a factor analysis based on the rule of thumb. Therefore, 333 samples were recruited for the CFA sample, including a 10% drop-out rate. The overall model fitness was determined using the “lavaan” package. The critical components of the selected fit indices were absolute fit, parsimony correction, and comparative fit [24,25]. Insignificant chi-square (P>0.05) and standardized root mean square residual (SRMR) and its 90% CI <0.08 were used to determine the absolute fit. Meanwhile, the criteria for parsimony correction fitness were RMSEA 0.08. Comparative fit index (CFI) and Tucker-Lewis’s index (TLI) values of 0.90 or higher were required for comparative fit. Factor loading selection was the same as EFA, which was greater than or equal to 0.30 [22] to be retained in the model. A factor correlation below 0.85 was deemed necessary, following the suggestion of Brown [24], demonstrating that the factors are distinct and that the model has no multicollinearity problem. To generate the final model, a model-to-model comparison was performed by comparing the Akaike Information Criterion (AIC) and Bayesian Information Criterion (BIC) in addition to the chi-square difference significance. AIC and BIC improvements with a significant p-value for the chi-square difference suggest that the final model was improved with the amendment [24]. Hence the final model was accepted for the next stage of the study.

iv) Reliability

The internal consistency (IC) of the knowledge domain items was measured using Cronbach's alpha coefficient. If the overall Cronbach's alpha value of the questionnaire items was greater than 0.70, the items were considered acceptable internal consistency [26]. Meanwhile, Raykov's rho value was used for composite reliability indices in the awareness and practice domains. It considers correlated errors. A composite reliability of 0.70 is acceptable [27].

## Results

Stage I: items development and content and face validity

An extensive literature review on scuba divers' safety and emergency response ideas resulted in developing a questionnaire's essential items and domains. Expert discussions during the content validation process refined the content by identifying relevant items and domains related to the scope of the study. All the items in the first version questionnaire scored more than 80% for I-CVI, S-CVI, and S-CVI/AVE by all experts. A few minor modifications were made regarding the usage of the words and terminologies without affecting the content of the questions. The second version was later tested on the intended participants for face validation. Based on their opinions, the wordings and terminologies were mainly clear and easy to understand, with minimal confusion. As a result, all the items in the questionnaire were scored >80% for I-FVI by the participants. The third version of the questionnaire set for validation comprises 25 items capturing sociodemographic characteristics and diving profile information. Additionally, it includes 21 items for the knowledge domain, and 16 for both awareness and practice domains, assessing a comprehensive assessment of scuba divers' KAP on safety and emergency response.

Stage II: questionnaire validation

i) Descriptive Analysis

For the overall validation stage, 555 participants were recruited, 222 participants for EFA and IRT and 333 participants for CFA. The participants' mean age was 34.50 years. Most participants were males (77.5%) and Malaysians (98.6%). Most participants belonged to Sabah native ethnicity (63.6%), including Dusun, Kadazan, Suluk, and Bajau who were coded as ‘others’. Additionally, 52.8% of the participants were open-water divers and had completed less than 50 dives since their certification, as presented in Table 2.

**Table 2:** Characteristic of the scuba divers who participated in validation study, n=555 IRT= Item Response Theory; EFA = Exploratory Factor Analysis; CFA = Confirmatory Factor Analysis; SD = Standard Deviation; BMI = Body Mass Index; PADI = Professional Association of Diving Instructors; SSI = Scuba Schools International; TDI = Technical Diving International; NAUI = National Association of Underwater Instructors

Variables	IRT and EFA (n=222)			CFA (n=333)			Total (n=555)		
	Mean (SD)	Frequency	%	Mean (SD)	Frequency	%	Mean (SD)	Frequency	%
Sociodemographic									
Age	34.69 (5.67)			34.38 (6.27)			34.50 (5.99)		
Gender									
Male		156	70.3		274	82.3		430	77.5
Female		66	29.7		59	17.7		125	22.5
Nationalities									
Malaysian		217	97.7		330	99.1		547	98.6
Non-Malaysian		5	2.3		3	0.9		8	1.4
Ethnicity									
Malay		62	27.9		99	29.7		161	29.0
Chinese		21	9.5		13	3.9		34	6.1
Indian		3	1.4		4	1.2		7	1.3
Others		136	61.2		217	65.2		353	63.6
Marital status									
Single		115	51.8		146	43.8		261	47.0
Married		92	41.4		160	48.0		252	45.4
Divorced		15	6.8		27	8.1		42	7.6
Occupation									
Professional		124	55.8		162	48.6		286	51.5
Self-employed		75	33.8		111	33.3		186	33.5
Unemployed		8	3.6		21	6.3		29	5.2
Student		15	6.8		39	11.7		54	9.7
Education level									
Primary school		1	0.5		2	0.6		3	0.5
Secondary school		64	28.8		91	27.3		155	27.9
Degree		125	56.3		188	56.5		313	56.4
Postgraduates		32	14.4		52	15.6		84	15.1
None		0	0		0	0		0	0
Medical illness									
Cardiovascular disease		16	7.2		41	12.0		56	10.1
Respiratory disease		11	5.0		16	4.8		27	4.9
Endocrine disease		5	2.3		3	0.9		8	1.4
Musculoskeletal disease		15	6.8		25	7.5		40	7.2
Psychiatric disorder		2	0.9		8	2.4		10	1.8
None		173	77.9		241	72.4		414	74.6
Smoking status									
Smoking		49	22.1		57	17.1		106	19.1
Vaping		26	11.7		74	22.2		100	18.0
Both		9	4.1		17	5.1		26	4.7
No		138	62.2		185	55.6		323	58.2
Alcohol consumption									
Yes		143	64.4		94	28.2		173	31.2
No		79	35.6		239	71.8		382	68.8
BMI	25.35 (3.80)			24.55 (3.62)			24.87 (3.71)		
Diving profile									
Level of certification									
Open water		119	53.6		174	52.3		293	52.8
Advance open water		72	32.4		134	40.2		206	37.1
Rescue dive		18	8.1		18	5.4		36	6.5
Divemaster		13	5.9		7	2.1		20	3.6
Agency of certification									
PADI		192	86.5		260	78.1		452	81.4
SSI		21	9.5		49	14.7		70	12.6
TDI		4	1.8		9	2.7		13	2.3
NAUI		5	2.3		15	4.5		20	3.6
Diving experience									
<1 year		41	18.5		65	19.5		106	19.1
2-3 years		59	26.6		56	16.8		115	20.7
4-5 years		44	19.8		77	23.1		121	21.8
6-10 years		62	27.9		111	33.3		173	31.2
>10 years		16	7.2		24	7.2		40	7.2
Frequency of dive since certificate granted									
<50 dives		96	43.2		164	49.2		260	46.8
50-100 dives		50	22.5		81	24.3		131	23.6
101-500 dives		62	27.9		67	20.1		129	23.2
>500 dives		14	6.3		21	6.3		35	6.3
Type of equipment used									
Own		56	25.2		106	31.8		162	29.2
Rental		120	54.1		125	37.5		245	44.1
Mixed		46	20.7		102	30.6		148	26.7
Type of checklist used									
Written		16	7.2		89	73.3		105	18.9
Remembered		206	92.8		244	73.3		450	81.8
Diving depth (Meters)									
≤18 (60 feet)		103	46.4		137	41.1		240	43.2
19-30 (61-99 feet)		102	45.9		151	45.3		253	45.6
>30 (100 feet)		17	7.7		45	13.5		62	11.2
Dive log									
Yes		87	39.2		184	55.3		271	48.8
No		135	60.8		149	44.7		284	51.2
Dive buddy									
Yes		216	97.3		327	98.2		543	97.8
No		6	2.7		6	1.8		12	2.2
Presence of mishaps									
Yes		100	45.0		160	48.0		260	46.8
No		122	55.0		173	52.0		295	53.2
Experience with dive-related injury/illness									
Yes		35	84.2		106	31.8		141	25.4
No		187	84.2		227	68.2		414	74.6

ii) Item Response Theory

As shown in Table 3, IRT was used to evaluate the psychometric properties of the knowledge domain of the questionnaire. Subdomains included safety-related items (KS1 to KS11) and emergency response items (KE1 to KE10). Items KS1, KS4, KS5, KS9, KE1, KE4, KE5, KE6, and KE10 were categorized as easy. KS7, KS8, KS10, KS11, KE8, and KE9 were deemed average, while the remaining items were considered hard. Only KS2, KS8, KS10, KS11, KE4, KE8, and KE9 fell within the -3 to +3 difficulty range. For discrimination levels, items KS5, KS7, KS8, KS9, KS10, KS11, KE1, KE4, KE8, and KE9 exhibited good discrimination.

**Table 3:** Result of Item Response Theory (IRT) analysis for knowledge domain (n=222) The modified parallel analysis supported multidimensionality with an RMSEA of 0.02 (95%CI:0.03, 0.06) χ2= chi square; df= Degree of Freedom; RMSEA= Root Mean Square Error of Approximation; CI= Confidence Interval

Item	Difficulty (b)	Discrimination (a)	χ2 (df = 8)	p-values
KS1: A scuba diver must follow the dive guide's / local guide briefing on where to go, the route, and what to watch out for before each diving.	-7.97	0.46	8.17	0.42
KS2: A Refresher course is recommended after an inactivity period of more than six months.	2.06	-15.13	14.89	0.06
KS3: When diving at depths greater than 10 meters, it is required to make a safety stop at a depth of 5 meters (15 feet) for at least three minutes to prevent decompression sickness.	6.05	-0.83	5.42	0.71
KS4: During descending, scuba divers need to regularly equalize their ears before they feel discomfort using the three maneuvers.	-10.70	0.45	2.15	0.98
KS5: Never hold your breath during diving to prevent lung overexpansion.	-4.04	1.24	11.72	0.16
KS6: Using complete, well-maintained, and reliable equipment that the scuba divers are familiar with can help to avoid dive accidents caused by equipment failure.	6.05	-0.83	2.15	0.98
KS7: Cleaning the equipment thoroughly after each dive can help to keep it in good condition and not rusty.	-1.88	0.87	14.37	0.07
KS8: Changes in the volume, density, and pressure of the gas are according to the depth of water	-1.61	4.41	10.88	0.21
KS9: At increasing depths, the partial pressure of nitrogen increases, causing narcosis in all divers.	-5.00	0.82	8.56	0.38
KS10: The deeper the scuba divers dive, the shorter the duration of being underwater.	-1.84	1.65	12.43	0.13
KS11: If the scuba divers ascend rapidly, nitrogen can form air bubbles and block blood flow, causing decompression sickness.	-0.88	0.90	49.23	<0.001
KE1: The higher the nitrogen concentration in the bloodstream, the slower the nervous system will be.	-3.57	1.25	13.99	0.08
KE2: Shivering is one of the symptoms that occurs when the body temperature decreases.	33.57	-0.13	7.27	0.51
KE3: When scuba divers get panicked and overwhelmed underwater, hyperventilation syndrome can occur.	6.73	-0.67	8.32	0.40
KE4: The eardrum can be injured if the scuba divers don’t equalize their ears regularly	-2.24	1.94	7.88	0.45
KE5: Early treatment of decompression injury begins with early recognition of the symptoms.	-3.73	0.70	15.63	0.05
KE6: Breathing pure oxygen may relieve decompression illness symptoms and should be given as soon as possible.	-35.67	0.10	11.40	0.18
KE7: It is imperative that dive centers always stock and keep their first aid kit up to date.	5.46	-0.94	5.53	0.70
KE8: If my dive buddy developed shortness of breath after diving, seeking help was the first action to be done.	-0.74	1.98	19.35	0.01
KE9: Every dive center must have an emergency response plan in situ.	-0.46	2.39	43.70	<0.001
KE10: In diving activities, having a personal emergency plan in place is essential to ensure that divers will respond appropriately in the event of an emergency.	-8.86	0.11	82.01	<0.001

Despite the varying difficulty and discrimination levels, item selection for the questionnaire was guided by item-fit analysis in the 2PL-IRT model to ensure a suitable fit. Based on the item-fit statistic, four items (KS11, KE8, KE9, KE10) showed poor fit for the 2PL-IRT model. Nevertheless, KE10 was excluded due to its low difficulty level and inadequate discrimination level, according to Baker [10] and Wyse [11]. Although KS11, KE8, and KE9 exhibited lower item fitness levels, they were retained based on their acceptable difficulty and satisfactory discrimination levels. Moreover, these items hold significance in assessing emergency response plan knowledge, as affirmed by expert input. This approach guaranteed that the questionnaire included relevant and meaningful items while considering the model's psychometric qualities.

The multidimensionality assumption of the items was supported by the modified parallel analysis with RMSEA of 0.02 (95%CI=0.005, 0.03), suggesting a good fit of the model to the data.

In conclusion, the knowledge domain of the questionnaire consists of two main subdomains: safety, with 11 questions (KS1 to KS11), and emergency response, with nine questions (KE1 to KE9). These items in the subdomains could accurately measure participants' comprehension of safety and emergency response in the context of scuba diving. 

iii) Exploratory Factor Analysis

For the awareness and practice domain, the items were subjected to EFA to examine the KMO test and Bartlett's test of sphericity to evaluate the factorability. The awareness domain comprises two initial factors: safety with 10 items (AS1 to AS10) and emergency response with six items (AE1 to AE6). The KMO test was 0.83, and the significance of Bartlett's test of sphericity was less than 0.001, indicating that the EFA was appropriate for the data following the EFA. All components displaying factor loadings exceeding 0.30 were retained in the model, except for AS2, AS9, AS10, and AE5. Consequently, these specific items were excluded from the analysis. The initial two factors were restructured into three to improve the model fitness, each with at least three items. The goal was to achieve an SRMR of 0.07, ensure no cross-loadings, and ensure that each item had sufficient communalities. Additionally, the factors have no significant correlations among them. Factor 1 was renamed "safety procedures" (AS1, AS3, AS4, AS5), Factor 2 was renamed "environmental hazards" (AS6, AS7, AS8), and Factor 3 was called "emergency response plan" (AE1, AE2, AE3, AE4, AE6) as shown in Table 4. These elements indicate the key themes for the questionnaire's awareness domain.

**Table 4:** Results of the Exploratory Factor Analysis (EFA) of the awareness domain Primary axis factoring was used for factor extraction. Oblique Promax rotation method applied. Model fitness is achieved with SRMR2 of 0.07, no communalities, no items cross loading, and absence of high correlation between factors. CI= Confidence Interval; SRMR= Standardized Root Mean Square Residual

Factors	Items	Factor loading	Cronbach’s alpha for reliability (95%CI)
Safety procedure	AS1: Compliance with safety instructions is required; otherwise, safety may be jeopardized.	0.33	0.80 (0.76, 0.84)
	AS3: To avoid dive injury/illness, a systematic buddy check should be performed under the supervision of a dive guide/local guide.	0.67	
	AS4: Drinking enough water during the dive day is essential to keep us hydrated during the dive	0.65	
	AS5: Among divers, we constantly encourage and remind our dive buddies to abide by safety instructions during each diving activity.	0.78	
Environmental hazards	AS6: The dive guide/ local guide should thoroughly explain the environmental conditions and safety precautions distinctive to each dive site.	0.32	
	AS7: Safety is likely compromised if scuba divers do not adhere to the surrounding environmental risk during the dive.	0.91	
	AS8: If I don't want to get hurt or attacked during diving, I shouldn't disturb dangerous marine life by poking, touching, chasing or other hazardous activities.	0.48	
Emergency response plan	AE1: The availability of an emergency response plan in place is a criterion for selecting my dive center.	0.30	
	AE2: I will request that I be briefed on the emergency response plan to know my role and responsibilities in an emergency.	0.46	
	AE3: A systematic emergency response plan is essential for a dive center to be able to handle and respond to emergencies in and out of the water safely	0.94	
	AE4: The benefits of having a personal emergency plan outweigh the time and effort it takes to do so	0.68	
	AE6: I should have basic medical knowledge so that I’m able to perform basic treatment in an emergency if permitted.	0.36	

Meanwhile, for the practice domain, the initial factors consisted of two factors as well, which were safety (PS1 to PS10) and emergency response (PE1 to PE6). The data was suitable for EFA with a KMO value of 0.79 and significant Bartlett's test of sphericity (p<0.001). Three items (PS1, PS8, and PE5) were removed, and the EFA was used to reconstruct into four factors, improving model fitness with an SRMR value of 0.08. No significant correlations were observed, and communalities were acceptable. Two factors (factors 2 and 3) were combined to maintain the significance of subdomain relevance. The two factors were merged because the items showed significant grouping and shared similar characteristics. This consolidation did not affect the overall fitness of the final model. As a result, the practice domain was divided into three factors: Factor 1 included items PS2, PS3, PS4, and PS9, which focused on "safety procedures"; Factor 2 comprised items PS5, PS6, and PS7, cantered on the "buddy system"; and Factor 3 consisted of items PS10, PE1, PE2, PE3, PE4, and PE6, addressing "emergency response" as stated in Table 5.

**Table 5:** Results of the Exploratory Factor Analysis (EFA) of the practice domain Primary axis factoring was used for factor extraction. Oblique Promax rotation method applied. Model fitness is achieved with SRMR2 of 0.08, no communalities, no items cross loading, and absence of high correlation between factors. CI= Confidence Interval; SRMR= Standardized Root Mean Square Residual

Factors	Items	Factor loading	Cronbach’s alpha for reliability (95%CI)
Safety procedure	PS2: I let my dive guide or buddy know if I have a medical issue before starting the diving activity.	0.58	0.76 (0.72, 0.81)
	PS3: Before each dive, I did a pre-safety dive equipment check to ensure the equipment was in good condition.	0.85	
	PS4: If I had a doubt about my dive equipment, I immediately reported it to my dive guide or dive center	0.69	
	PS9: If I didn’t dive for more than six months, I’d do a refresher dive.	0.50	
Buddy system	PS5: Together, my dive buddy, dive guide, and I frequently talk about safety issues throughout the dive.	0.48	
	PS6: My dive buddy and I stayed close to each other and followed our dive guide underwater.	0.53	
	PS7: If in doubt, I did a buddy check more than once.	0.40	
Emergency response	PS10: I keep my safety knowledge up to date to ensure and promote safety.	0.32	
	PE1: I did take time to familiarize myself with the emergency response plan in each dive center I went to	0.73	
	PE2: I always ensure that my ability to respond appropriately in an accident is always adequate.	0.64	
	PE3: If I started to show signs of an injury or illness that could be related to diving, I alerted my dive guide or dive buddy right away.	0.95	
	PE4: If my dive buddy developed a medical complication such as shortness of breath, I did seek the nearest help.	0.44	
	PE6: I discussed with my guide or buddy an emergency response before starting the dive session	0.48	

iv) Confirmatory Factor Analysis

After completing the EFA and obtaining the final questionnaire, the subsequent validation process was carried out using CFA to determine how well the measured variables represent the number of components. Given that the data was not multivariate normal (kurtosis >5), MLR (robust maximum likelihood) analysis was chosen for the method of estimation [28].

CFA was conducted in the awareness domain on three factors with 12 items. Items AS1 and AE4, with low factor loadings of 0.24 and 0.25, were removed from the final model while retaining the rest of the items. Model 4 showed an insignificant chi-square, SRMR of 0.04, indicating a solid absolute fit. RMSEA was 0.035 (95% CI: 0.035, 0.001), implying a good parsimonious fit. CFI and TLI were high at 0.98 and 0.96, respectively, indicating solid comparative fit. The model comparison revealed improved AIC and BIC values, and significant chi-square differences, leading to model enhancements as outlined in Table 6.

**Table 6:** Fit indices of the models for awareness and practice domain χ2= Chi Square; df= Degree of Freedom; SRMR= Standardized Root Mean Square Residual; RMSEA= Root Mean Square Error of Approximation; CI= Confidence Interval; CFI=  Comparative Fit Index; TLI= Tucker-Lewis’s Index; AIC= Akaike Information Criterion; BIC= Bayesian Information Criterion

Domain	Model	χ2 (df)	p-value	χ2 diff (df)	p-value difference	SRMR	RMSEA (95% CI)	CFI	TLI	AIC	BIC
Awareness	Model Default	117.775 (51)	<0.001	29.6722 (10)	<0.001	0.07	0.062 (0.047, 0.077)	0.89	0.86	7945.80	8048.60
	Model1	86.764 (41)	<0.001	7.0556 (4)	0.13	0.06	0.057 (0.040, 0.074)	0.92	0.90	7332.60	7427.80
	Model2	56.597 (37)	0.03	1.8819 (2)	1.00	0.05	0.039 (0.014, 0.059)	0.97	0.95	7310.50	7420.90
	Model3	49.864 (35)	0.06	13.9796 (7)	0.05	0.04	0.035 (0.001, 0.056)	0.98	0.96	7307.70	7425.80
	Model4	39.532 (28)	0.08	-	-	0.04	0.035 (0.001, 0.059)	0.98	0.96	6860.30	6963.10
Practice	Model Default	285.058 (62)	<0.001	24.493 (11)	<0.05	0.07	0.101 (0.088, 0.115)	0.78	0.72	6816.10	6926.60
	Model1	269.742 (51)	<0.001	10.892 (7)	0.14	0.08	0.11 (0.096, 0.126)	0.78	0.71	6259.50	6362.40
	Model2	196.419 (44)	<0.05	100.482 (3)	<0.001	0.07	0.10 (0.085, 0.116)	0.84	0.77	6200.20	6329.70
	Model3	61.184 (41)	<0.05	-	-	0.04	0.04 (0.007, 0.056)	0.98	0.97	6071.00	6211.90

In the practice domain, the initial model featured three factors encompassing 13 items. Item PE2 was eliminated due to its low factor loading of 0.26. The final model exhibited a good absolute fit, as indicated by an SRMR of 0.04, despite a significant chi-square (p-value < 0.05). Both parsimonious and comparative fitness were good, with an RMSEA of 0.04 (95% CI: 0.007, 0.056), CFI of 0.98, and TLI of 0.97. Model-to-model comparison highlighted significant differences in chi-square values and improvements in AIC and BIC, underscoring the final model's improvement as outlined in Table 6.

In summary, the CFA results indicate that the awareness domain comprises three factors with 10 items, and the practice domain comprises three factors with 12 items, as in Table 7. These items and those from the knowledge domain will be utilized for the remaining stages of the study.

**Table 7:** Factor loadings and reliability of the final model.

Domain	Factor	Item	Factor loading	Raykov’s rho
Awareness	Factor 1: Awareness of safety procedure	AS3	0.53	0.71
		AS4	0.51	
		AS5	0.74	
	Factor 2: Awareness of environmental hazards	AS6	0.42	
		AS7	0.49	
		AS8	0.42	
	Factor 3: Awareness of emergency response	AE1	0.74	
		AE2	0.80	
		AE3	0.57	
		AE6	0.40	
Practice	Factor 1: Practice on safety procedure	PS2	0.87	0.55
		PS3	0.66	
		PS4	0.49	
		PS9	0.32	
	Factor 2: Practice on the buddy system	PS5	0.34	
		PS6	0.33	
		PS7	0.83	
	Factor 3: Practice on emergency response	PS10	0.37	
		PE1	0.47	
		PE3	0.54	
		PE4	0.38	
		PE6	0.34	

v) Reliability

In the knowledge domain, Cronbach’s alpha value is 0.77 for the entire domain, which is considered acceptable for assessing construct reliability. Meanwhile, the internal consistency reliability for the awareness and practice domains was acceptable in the EFA. The Cronbach’s alpha values were 0.80 for awareness and 0.78 for practice (Tables 4, 5).

However, differences in reliability indices emerged during the CFA. The composite reliability was deemed satisfactory for the awareness domain with a Raykov’s rho value of 0.71. On the other hand, the practice domain exhibited lower composite reliability, as indicated by Raykov’s rho value of 0.55, which falls below the acceptable threshold.

## Discussion

While safety modules are available for recreational scuba diving, revising and updating them periodically is crucial. Certifications are commonly seen as permanent credentials, often resulting in a dearth of renewal or re-examination processes. Notwithstanding this fact, injuries associated with diving persist, with ambiguous causes. The insufficiency of validated safety and emergency response questionnaires within the scuba diving community highlights the need for additional study. To address this disparity, our research conducted a comprehensive investigation by developing a validated survey instrument to assess the safety knowledge, attitudes, and behaviors of those engaged in scuba diving.

The initial questionnaire underwent expert validation and achieved a favorable score, with revisions made to improve the questions' content, clarity, and wording. The modifications were implemented to enhance the participants' understanding and mitigate the possibility of any perplexity. The face validation process demonstrated a significant level of clarity and comprehensibility, as evidenced by scores over 95% for each component. This indicates that participants provided outstanding responses to the questionnaire across various certification and education levels.

For the knowledge domain, IRT was chosen for data analysis due to its ability to provide deeper insights into the difficulty and discriminative nature of the questionnaire. At the same time, the 2PL-IRT model was the most suitable method for addressing the multiple true-false questions in the knowledge domain [29]. The item fitness determined the selection of the best item in the questionnaire. Four items (KS11, KE8, KE9, KE10) exhibited low item fitness; nevertheless, solely KE10 was excluded. Items KE8 and KE9 were included in the questionnaire to evaluate participants' understanding of the significance of emergency response plans within the recreational scuba diving sector. This aspect holds significant importance for the overall operations of dive centers, and it was deliberated upon during discussions with experts. Despite a few items showing undesirable difficulty levels and discrimination, we kept them in the questionnaire due to their alignment with item fitness criteria. This decision ensured a well-rounded range of perspectives, enhancing respondents' knowledge acquisition process. Few other studies suggested that the item with a low level of discrimination may cause problems in overall fitness [30]; However, in our study, the modified parallel analysis for multidimensionality showed a strong fit, confirming that the questionnaire's items effectively measure distinct dimensions that work together cohesively.

EFA was employed to assess the internal structure validity within the areas of awareness and practice. The questionnaire included items that had factor loadings of more than 0.3, per the recommendations of Comrey and Lee [9], Child [21] and Keenan and Stevens [20]. Subsequently, the factors were restructured to improve the model's fitness. The initial factor division of the practice domain resulted in four factors with a desirable model fitness. However, Factor 3 contained only two items, falling short of the acceptable threshold for items within a factor. Factors with only one or two salient loadings are expected to have low communalities, suggesting minimal explanatory power [9,24,31].

Based on the assessment of item relevance, a decision was made to combine one element with another. As a result, item PS9 was relocated to Factor 1, whereas item PE1 was allocated to Factor 3. The decision to merge the practice domain, particularly given the restricted number of items in Factor 3, was a well-justified choice. By combining factors with fewer items, we aimed to enhance model interpretability while maintaining conceptual relevance [24,32,33]. Merging was guided by shared content and was validated by experts, ensuring it didn't impact the final model's fitness. This practical and logical step was supported by expert consensus.

After assessing convergent and discriminant validity during EFA, the finalized construct underwent CFA to confirm the measurement theory. CFA validates, tests, and demonstrates the hypothesized factor structure explored in the EFA and serves as a crucial step to ensure the stability and generalizability of the factor structure [24]. Items with factor loading less than 0.3 didn’t belong to the factor and were decided to be eliminated from the construct as applied in EFA [20,21,34]. In our study, two items in the awareness domain (AS1 and AE4) and one item in the practice domain (PE2) were eliminated in the final construct. The model was improved statistically by removing the problematic items in each factor.

Moreover, the reliability of the knowledge domain in IRT was acceptable; the same goes for EFA for the awareness and practice domain. However, the composite reliability was 0.55, below the acceptable value of 0.7 for the CFA of the practice domain. The low reliability in our study has a few reasons. Initially, it was noted that several factor loadings observed in the practice items were below the recommended threshold of 0.5, as proposed by Awang [35]. This suggests that relatively weak associations between the items and the underlying variables exist. When the factor loadings exhibit low values, it implies that the items are insufficient in capturing the variance of the construct they are designed to assess, which can lead to lower consistency [36]. Factor loading cutoffs chosen were based on EFA limits. Utilizing the same cutoff value for EFA and CFA ensures consistent factor loading interpretation, allowing a seamless transition from data exploration to confirmation of the hypothesized structure. Extending the cutoff to CFA indicates adherence to the same conceptual framework, model alignment and factor structure stability.

Additionally, our final model had a small number of items, and some factors needed more items, affecting reliability. Smaller items limit variability in response, which depends on the variance in the data, affecting reliability estimates and measurement specificity. They may not capture participant variability or cover the full breadth and depth of the measured construct [37-40].

Given the distinctive characteristics of our internal validity results and a scarcity of observed reliability value, we have considered this value a satisfactory pragmatic compromise for assessing the reliability of our measurement instrument. The conclusion is substantiated by the strong evidence from CFA, which confirms the validity of the constructs and is also consistent with the overall research goals.

Our study has limitations as it focused exclusively on diving centers in East Malaysia, potentially not fully representing the entire diving community in the country. A more comprehensive approach would involve collaboration with dive agencies and associations to recruit participants nationwide. Additionally, the use of universal sampling may exclude specific subgroups, leading to potential biases. Future research should broaden the study's scope by employing multi-stage random sampling and adaptable sample preparation methods to enhance generalizability across diverse populations.

## Conclusions

In summary, our study aimed to develop and validate a questionnaire for assessing scuba divers' knowledge, awareness, and practice regarding safety and emergency response. We used IRT, EFA, and CFA for validation. The final questionnaire includes a demographic checklist, diving profile info, and three main KAP sections. The knowledge domain contains 19 items, awareness has 10, and practice has 12. The questionnaire showed good psychometric properties and reliability, except for practice, which had lower reliability. Enhancements in the future, like more items and a larger sample size, could improve the practice domain's reliability, addressing our study's limitations.
